# Treatment of obstructive sleep apnea with CPAP improves daytime sleepiness and fatigue in cancer patients

**DOI:** 10.1002/cam4.7198

**Published:** 2024-10-30

**Authors:** Kimia G. Ganjaei, Karen A. Wong, Shiela M. Strauss, Sigrid V. Carlsson, Margaret Barton‐Burke, Miranda Tan

**Affiliations:** ^1^ Pulmonary Service, Department of Medicine Memorial Sloan Kettering Cancer Center New York New York USA; ^2^ NYU Rory Meyers College of Nursing New York New York USA; ^3^ Department of Nursing Research Memorial Sloan Kettering Cancer Center New York New York USA; ^4^ Department of Epidemiology and Biostatistics Memorial Sloan Kettering Cancer Center New York New York USA; ^5^ Department of Surgery Memorial Sloan Kettering Cancer Center New York New York USA; ^6^ Department of Urology, Institute of Clinical Sciences Sahlgrenska Academy at University of Gothenburg Gothenburg Sweden; ^7^ Division of Sleep Medicine, Department of Psychiatry Stanford University School of Medicine Palo Alto California USA

**Keywords:** cancer, fatigue, obstructive sleep apnea, pap therapy, sleepiness

## Abstract

**Background:**

Fatigue and sleep disorders are prevalent in cancer patients. Obstructive sleep apnea (OSA) commonly causes excessive daytime sleepiness (EDS) and fatigue. We hypothesize that treating cancer patients with OSA using positive airway pressure (PAP) will improve EDS and fatigue.

**Methods:**

A retrospective chart review of sleep clinic visits of cancer patients with newly diagnosed OSA was performed. Epworth Sleepiness Scale (ESS) and fatigue reported at baseline and within 6 months of starting PAP therapy were compared between PAP‐adherent and PAP‐non‐adherent patients.

**Results:**

65 cancer patients with OSA and ESS >10 were recommended PAP therapy, including 45 patients with fatigue. 29 patients pursued PAP therapy with 79% (*n* = 23) adherent at follow‐up. The median baseline apnea hypopnea index (AHI) for OSA was 24.0 (interquartile range [IQR] 14.3, 32.3) and 23.8 (IQR 10.1, 42.8) events/hour among PAP‐adherent and PAP‐non‐adherent patients, respectively (*p* = 0.90). Median baseline ESS was 14.0 (IQR 12.0, 17.0) among adherent and 17.0 (IQR 11.0, 17.3) among non‐adherent patients (*p* = 0.73). The median ESS at follow‐up of the adherent and non‐adherent groups was 8.0 (IQR 6.0, 10.0) and 11.0 (IQR 8.0, 15.8), respectively (*p* = 0.08). Median ESS change was −5.0 (IQR −7.0, −4.0) in PAP adherent patients and −2.5 (IQR −5.25, −1.50) in PAP‐non‐adherent patients (*p* = 0.07). When the groups are examined separately, the median change in the PAP‐adherent group was highly significant (*p* = 0.001), while the ESS median change in the PAP‐non‐adherent group was considerably less (*p* = 0.04). 17 out of the 21 PAP‐adherent patients reporting fatigue at baseline indicated improvement at follow‐up.

**Conclusions:**

PAP therapy for OSA in cancer patients improves EDS and fatigue. Larger studies are necessary to evaluate the efficacy of PAP in improving fatigue in this population.

## 1 INTRODUCTION

Fatigue and sleep disturbances are common in cancer patients, many of whom continue to have ongoing difficulty with sleep after treatment.^1^, ^2^, ^3^ The National Comprehensive Care Network recognizes sleep disturbance as one of the primary factors associated with cancer‐related fatigue and recommends management of sleep disturbances.^4^ Obstructive sleep apnea (OSA), a highly prevalent and treatable sleep disorder,^5^, ^6^ is not usually evaluated in cancer patients. Common symptoms of OSA include daytime sleepiness, fatigue, and unrefreshing sleep.^7^, ^8^ Many cancer patients have risk factors for OSA, but this is not always identified prior to initiating cancer therapy, and their fatigue may be attributed to the cancer itself or treatment‐related side effects.^3^, ^9^ Additionally, some cancer patients develop OSA as a result of their specific type of cancer or treatment, such as those with head and neck tumors.^10^, ^11^, ^12^

OSA is characterized by recurrent upper airway obstruction during sleep that may cause reduction or cessation of airflow, intermittent hypoxia (IH), and sleep fragmentation. OSA‐related IH has been implicated in tumor development and progression. IH in animal models has demonstrated upregulation of inflammatory markers (transforming growth factor‐beta, vascular endothelial growth factor) and hypoxia‐related pathways, which was associated with increased fibroblast proliferation, cell migration,^13^ and tumor growth.^14^, ^15^ IH from OSA also activates the transcription factor hypoxia‐inducible factor (HIF). HIF isoforms are expressed in a wide array of cancers and contribute to tumor progression following activation by IH.^16^, ^17^

While there is evidence of high prevalence of OSA in cancer patients,^18^, ^19^, ^20^ the literature on treating OSA in these patients to improve daytime sleepiness and fatigue is quite limited. The gold standard treatment for OSA is positive airway pressure (PAP) therapy.^6^ In this study, we examine PAP for treatment of newly diagnosed OSA in a cancer population. We hypothesize that its use will improve excessive daytime sleepiness (EDS) and fatigue.

## 2 METHODS

### 2.1 Study population

The study was approved by the Memorial Sloan Kettering Institutional Review Board in 2020. A retrospective chart review was conducted of cancer patients who were evaluated in sleep clinics at Memorial Sloan Kettering Cancer Center from 2019 to 2020 for EDS and fatigue within 1 month of a new OSA diagnosis. A waiver of informed consent was granted by the IRB due to the retrospective nature of the study. OSA was diagnosed with a home sleep apnea test (HSAT) or in‐lab polysomnogram (PSG).

### 2.2 Inclusion and exclusion criteria

Patients aged 18 and older with a new diagnosis of OSA were included if they had an initial Epworth Sleepiness Scale (ESS) score >10, fatigue at the time of evaluation, and were planning to initiate PAP therapy. Patients were excluded if they had no sleep study available, had another sleep disorder present, or were trying an alternative therapy for OSA. Patients had follow‐up within 6 months to reassess EDS and fatigue in the sleep clinic after initiation of PAP therapy.

### 2.3 Data collection

Data that were collected from chart review at the time of the sleep study included age, gender, body mass index (BMI), type of cancer, stage of cancer, responses to the STOP‐Bang questionnaire^21^ (presence of snoring, witnessed apneas, neck size, tiredness, etc.), and prescription sedatives. Comorbidities included were obesity, hypertension, diabetes, chronic obstructive pulmonary disease, and asthma.

### 2.4 Measures

All sleep studies were scored using the Center for Medicare and Medicaid Services (CMS) scoring criteria.^22^ Apnea was defined as complete (>90%) reduction in airflow lasting ≥10 seconds. A hypopnea was scored if there was partial (≥30%) reduction in airflow lasting ≥10 seconds accompanied by a 4% arterial oxygen desaturation from baseline. The apnea‐hypopnea index (AHI) was recorded to define the severity of OSA. AHI ≥5 events/hour was defined as OSA; AHI 5–14, 15–29, and ≥30 events/hour were classified as mild, moderate, and severe OSA, respectively.

EDS was defined as ESS >10 and assessed using the ESS at baseline and at follow‐up within 6 months of PAP initiation. PAP adherence data were reviewed at the time of follow‐up. PAP adherence was defined using the Centers for Medicare Services (CMS) criteria, which requires usage of PAP greater than or equal to 4 hours per night on 70% of nights during a period of 30 consecutive days.^22^

The ESS is a validated questionnaire often used for clinical assessment in sleep medicine that evaluates the likelihood of falling asleep in a series of eight common situations. Each activity is rated on a four‐point Likert scale of 0–3, with 3 representing the highest chance of falling asleep. This instrument has been used to measure sleepiness in various populations.^23^

The presence of fatigue at baseline was determined by self‐report of fatigue or by the answer of ‘yes’ to the question “do you often feel tired, fatigued, or sleepy during the daytime?” (i.e., ‘T' in the STOP‐Bang questionnaire) at initial sleep visit prior to the sleep study. The STOP‐Bang questionnaire was used because it was consistently available in clinical sleep notes for assessment of fatigue as opposed to other surveys. The STOP‐Bang questionnaire is a validated survey that screens for the likelihood that a patient has undiagnosed OSA.^21^ A total score <3 confers a low risk of OSA. Scores of 3–4 and 5–8 indicate an intermediate and high risk of OSA, respectively. Patients were asked whether fatigue was present, improved, or not present at the follow‐up visit within 6 months of initiating PAP treatment for OSA.

### 2.5 Data analysis

Statistical analyses were performed using SPSS version 26. Proportions, medians, and 25th–75th percentiles described the characteristics of our cancer population. ESS was compared between PAP‐adherent and PAP‐non‐adherent participants using Mann–Whitney *U* tests. Fatigue and other patient characteristics were compared between PAP‐adherent and PAP‐non‐adherent groups using Fisher's exact tests and Mann–Whitney *U* tests. Within‐group ESS change from baseline to follow‐up was evaluated with the Wilcoxon Matched‐Pairs test. A *p*‐value of 0.05 or less was considered statistically significant.

## 3 RESULTS

A total of 65 cancer patients with newly diagnosed OSA with an ESS >10 was identified. Within this group, 69% (*n* = 45) reported fatigue at baseline. Demographics of this group of 45 individuals are shown in Table 1. The median baseline AHI of our cohort fell into the moderate range at 20.3 and median baseline ESS was 14 (Table 2).

**TABLE 1:** Demographics of persons eligible for the study who reported fatigue at baseline.

Characteristics	*n*	%	Md	(Q1, Q3)
Gender				
Male	26	57.8		
Female	19	42.2		
Age			60.0	(50.0, 67.5)
BMI^a^			32.4	(28.1, 34.925)
Cancer type^a^				
Breast	8	19.0		
Colon/Rectal	4	9.5		
Hematologic	3	7.1		
Lung	3	7.1		
Prostate	9	21.4		
Renal	3	7.1		
Other	12	28.6		
Metastatic cancer	2	4.4		
Comorbid conditions				
Diabetes	6	13.3		
COPD	6	13.3		
HTN	30	66.7		
Asthma	9	20.0		
Use of sedatives	4	8.9		

Abbreviations: BMI, body mass index; COPD, chronic obstructive pulmonary disease.

^a^ *N* = 42.

**TABLE 2:** Symptoms and OSA features of persons eligible for the study who reported fatigue at baseline.

Characteristics	*n*	%	Md	(Q1, Q3)
Self‐reported STOP‐Bang characteristics				
Snoring	36	80.0		
Observed apnea	22	48.9		
Daytime somnolence	43	95.6		
HTN	27	60.0		
BMI > 35^a^	10	22.2		
Age > 50	34	75.6		
Neck circumference >17	26	57.8		
STOP‐Bang score			5.0	(4.0, 6.0)
Sleep study information				
Type of study				
HSAT	34	75.5		
PSG	11	24.5		
Baseline AHI			20.3	(10.55, 32.65)
O_2_ nadir			80.0	(75.0, 83.0)
Baseline fatigue			100.0	(100.0, 100.0)
Baseline ESS			14.0	(12.0, 17.0)

*Note*: *N* = 45.

Abbreviations: AHI, apnea hypopnea index; BMI, body mass index; ESS, Epworth Sleepiness Scale; HSAT, home sleep apnea test; Md, median; OSA, obstructive sleep apnea; PSG, in‐lab polysomnogram; Q1, 25th percentile; Q3, 75th percentile.

^a^ *N* = 42.

In the fatigued group, 64% accepted prescribed PAP therapy and returned for follow‐up. Of these, 79% (*n* = 23) were adherent with PAP therapy at follow‐up, which occurred on average at 3 months post‐baseline. The adherent and non‐adherent groups were not significantly different in terms of clinical characteristics, AHI, or fatigue and sleepiness scores (Table 3). The median baseline ESS was 14.0 (interquartile range [IQR] 12.0, 17.0) among PAP‐adherent and 17.0 (IQR 11.0, 17.3) among the PAP‐non‐adherent group (*p* = 0.73). The median baseline AHI at the time of the sleep study was 24.0 (14.3, 32.3) events/hour for the PAP‐adherent group and 23.8 (10.1, 42.8) events/hour for the PAP‐non‐adherent group (*p* = 0.90).

**TABLE 3:** Characteristics and OSA features of persons eligible for the study who reported fatigue at baseline and had follow‐up.

Baseline characteristic	Full sample		PAP‐adherent		PAP‐non‐adherent		*p* ^b^
	*n*	%	*n*	%	*n*	%	
Gender							0.646
Male	18	62.1	15	65.2	3	50.0	
Female	11	37.9	8	34.8	3	50.0	
Comorbid conditions							
Diabetes	3	10.3	3	13.0	0	0.0	1.000
COPD	5	17.2	3	13.0	2	33.3	0.269
HTN	20	69.0	17	73.9	3	50.0	0.339
Asthma	6	20.7	4	17.4	2	33.3	0.575
Self‐reported STOP‐Bang characteristics							
Snoring	23	79.3	19	82.6	4	66.7	0.575
Observed apnea	16	55.2	12	52.2	4	66.7	0.663
Daytime tiredness, fatigue, or somnolence	27	93.1	22	95.7	5	83.3	0.377
HTN	18	62.1	15	65.2	3	50.0	0.646
BMI > 35^a^	5	17.2	3	13.0	2	33.3	0.303
Age > 50	23	79.3	19	82.6	4	66.7	0.575
Neck circumference >17 in	13	44.8	11	47.8	2	33.3	0.385
Type of sleep study							1.000
HSAT	19	65.5	15	65.2	4	66.7	
PSG	10	34.5	8	34.8	2	33.3	

Baseline characteristic	Full sample		PAP‐adherent		PAP‐non‐adherent		*U*	*Z*	*p* ^c^
	Md	(Q1, Q3)	Md	(Q1, Q3)	Md	(Q1, Q3)			
Age	64.0	(53.5, 69.5)	64.0	(56.0, 71.0)	61.0	(43.8, 67.3)	47.0	−1.187	0.254
BMI	29.6	(27.3, 34.2)	29.6	(27.9, 33.4)	30.3	(23.7, 40.3)	58.0	−0.122	0.929
O_2_ nadir	81.0	(75.0, 83.0)	80.0	(75.0, 83.0)	82.5	(77.3, 84.5)	52.5	−0.892	0.384
STOP‐Bang Score	5.0	(4.0, 6.0)	5.0	(4.0, 6.0)	4.5	(3.75, 6.0)	52.0	−0.944	0.384
AHI Baseline	24.0	(11.5, 34.3)	24.0	(14.3, 32.3)	23.8	(10.1, 42.8)	66.0	−0.162	0.896
ESS Baseline	14.0	(11.5, 17.0)	14.0	(12.0, 17.0)	17.0	(11.0, 17.3)	62.0	−0.381	00.733

*Note*: *N* = 29.

Abbreviations: AHI, apnea‐hypopnea index; BMI, body mass index; COPD, chronic obstructive pulmonary disease; ESS, Epworth Sleepiness Scale; HSAT, home sleep apnea test; HTN, hypertension; Md, median; PSG, in‐lab polysomnogram; Q1, 25th percentile; Q3, 75th percentile.

^a^ *N* = 27.

^b^ Fisher's exact test.

^c^ Mann–Whitney *U* test.

Comparing PAP‐adherent and PAP‐non‐adherent groups, the median ESS at follow‐up was 8.0 (IQR 6.0, 10.0) and 11.0 (IQR 8.0, 15.8) in the PAP‐adherent and PAP‐non‐adherent groups respectively (*p* = 0.08), and median ESS change was −5.0 (IQR −7.0, −4.0) and −2.5 (IQR −5.25, −1.50) in the PAP‐adherent and PAP‐non‐adherent groups respectively (*p* = 0.07) (Table 4). When the groups are examined separately, the median change of −5.0 (IQR −7.0, −4.0) in the PAP‐adherent group was highly significant (*p* = 0.001), while the ESS median change of −2.5 (IQR −5.25, −1.50) was considerably less so in the PAP‐non‐adherent group (*p* = 0.04).

**TABLE 4:** ESS characteristics of persons eligible for the study who reported fatigue at baseline and had a follow‐up.

Characteristic	Full sample		PAP‐adherent		PAP‐non‐adherent		*U*	*Z*	*p* ^a^
	Md	(Q1, Q3)	Md	(Q1, Q3)	Md	(Q1, Q3)			
ESS baseline	14.0	(11.5, 17.0)	14.0	(12.0, 17.0)	17.0	(11.0, 17.3)	62.0	−0.381	0.733
ESS follow‐up	8.0	(7.0, 10.5)	8.0	(6.0, 10.0)	11.0	(8.0, 15.8)	22.5	−1.767	0.08
ESS change	−4.0	(−6.5, −3.0)	−5.0	(−7.0, −4.0)	−2.5	(−5.25, −1.50)	21.0	−1.882	0.07

*Note*: *N* = 29. Using the Wilcoxon Matched‐Pairs test, in the PAP‐adherent group, there was a statistically significant median ESS change from baseline to follow‐up of −5.0 (−7.0, −4.0), *Z* = −3.305, *p* = 0.001, and in the PAP‐non‐compliant group there was a much less pronounced ESS change from baseline to follow‐up of −2.5 (−5.25, −1.50), *p* = 0.04.

Abbreviations: ESS, Epworth Sleepiness Scale; Md, median; Q1, 25th percentile; Q3, 75th percentile.

^a^ Mann–Whitney *U* test.

Of the 23 PAP‐adherent patients, 17 (74%) reported that fatigue improved or was no longer present at follow‐up, 4 reported no change, and 2 did not have documentation of fatigue level.

## 4 DISCUSSION

Cancer patients frequently report fatigue, which is often attributed to disease and its associated therapies. Clinicians need to consider OSA in the evaluation of sleep disturbance in cancer patients just as they would in the general population, and perhaps with even greater urgency in the setting of treatments that can exacerbate fatigue.^1^, ^3^ In our population of cancer patients with a variety of cancer types with newly diagnosed OSA, adherence to PAP therapy was found to improve daytime sleepiness and fatigue.

OSA treatment in the general population is associated with many symptomatic benefits including improvement in EDS,^24^, ^25^ fatigue,^24^ quality of life measures,^25^ and sleep quality rating^26^; these measures are associated concurrently with a reduction in OSA severity and AHI even over a short treatment period of 3^24^ to 4 weeks.^25^ Our population had a moderate median AHI and adherent patients reported significant improvement in fatigue, suggesting the potential profound effect of PAP therapy for cancer patients with even mild to moderate OSA. There are likely to be additional benefits of PAP therapy for cancer patients with OSA as there are in the general population. While our study did not specifically examine functional status, PAP therapy for OSA patients has been associated with improved exercise duration,^27^ cognition,^28^ memory tasks,^29^ as well as reduced depression,^28^, ^30^ fewer motor‐vehicle accidents or near‐miss accidents,^31^, ^32^ and less pain.^33^

There is a potential association between OSA—particularly when untreated—on cancer prognosis in a variety of cancers including colorectal cancer, melanoma, and lung cancer.^34^, ^35^, ^36^, ^37^ In a small study of the serum from lung cancer patients, OSA was associated with T‐cell downregulation and increased expression of inflammatory markers.^38^ These serological markers were associated with worse short‐term survival, though this included both patients with and without OSA.^38^ Severe OSA has been associated with worse prognosis in a small group of patients with stage III and IV lung cancer.^36^ Not surprisingly, when including participants with lung cancer at various stages, an association between OSA and mortality was not found.^39^ This may be due to the inherently poor prognosis for many patients with advanced stage cancers regardless of OSA diagnosis and reflects one of the challenges of studying OSA in this population.

PAP therapy may also promote disease control in patients with OSA and cancer. In a study of patients with melanoma, patients with OSA adherent to PAP therapy had lower rates of metastatic disease and all‐cause mortality on 5‐year follow‐up compared with patients who had untreated OSA.^37^ In fact, PAP‐compliant patients in this study had similar survival to those without OSA. It is difficult to overstate the value of disease control in improving the quality of life in cancer patients, particularly when comorbid conditions such as OSA are treatable.

There were a few limitations in this study. Even though the study was powered to evaluate for a difference in the primary outcome, the small sample size limited the ability to perform subgroup analyses. The small size of the study and lower follow‐up in the PAP‐non‐adherent group is likely why there was only a trend to significance in follow‐up ESS score differences between PAP‐adherent and PAP‐non‐adherent patients. Low follow‐up in the PAP‐non‐adherent group may have been due to the perceived limited purpose of a follow‐up visit for the patients who were not using PAP therapy, or potentially due to complications of cancer. The statistically significant improvement in fatigue and ESS in the PAP‐adherent group supports the fact that treating OSA in cancer patients could improve fatigue and EDS initially ascribed to other causes. The unexpected smaller, though statistically significant improvement in ESS in the PAP‐non‐adherent group could reflect improved daytime sleepiness related to non‐OSA factors such as cancer treatment. This is an important finding for future studies because the cancer population has multiple self‐perceived contributing factors to sleepiness and fatigue, and this may limit the ability to detect differences between PAP‐adherent and PAP‐non‐adherent groups in small studies.

Given the retrospective nature of the study, baseline assessment of fatigue was limited to self‐report and the STOP‐Bang questionnaire, which has been consistently used in the clinical setting. Additional surveys of the degree of fatigue and related functional limitations could provide insight on the quality of life of cancer patients with OSA in future studies. However, fatigue surveys need to be evaluated for relevance in the OSA population. Assessment of PAP adherence was limited to the percentage of patients who met CMS criteria because it was felt to be more accurate and pertinent than using the raw data of hours of PAP use per night and residual AHI. This was because of short‐term follow‐up, small sample size, and quantitative details were not consistently provided given the nature of the retrospective study. Additional data on hours of CPAP use and residual AHI may be beneficial for larger prospective studies of OSA in cancer patients.

There are several challenges in studying OSA in the cancer population. It is important to carefully choose quality of life and functional status measures which are expected to change with correction of OSA and compare an individual's degree of improvement compared to baseline. Even when studying specific types of cancers, the population can be quite heterogenous in terms of age, baseline functional status, and especially cancer staging. To understand effects on cancer prognosis in addition to quality of life, longer follow‐up is needed, and this can be challenging, particularly in patients with advanced stage cancer.

## 5 CONCLUSIONS

PAP therapy for OSA improves fatigue and EDS in cancer patients with OSA. Clinicians should consider evaluation for OSA in cancer patients with fatigue and EDS, particularly with ESS scores greater than 10 and appropriate risk factors. Larger studies are necessary to determine the effect of PAP in improving fatigue and functional status for cancer patients with OSA and EDS. Given that OSA is treatable and can significantly impact quality of life of cancer patients, it should be recognized and treated appropriately with PAP therapy without delay.

## AUTHOR CONTRIBUTIONS

**Kimia G. Ganjaei:** Visualization (equal); writing – original draft (equal); writing – review and editing (equal). **Karen A. Wong:** Writing – original draft (equal). **Shiela M. Strauss:** Formal analysis (equal); investigation (equal); resources (equal); validation (equal); visualization (equal); writing – review and editing (equal). **Sigrid V. Carlsson:** Formal analysis (equal); investigation (equal); visualization (equal). **Margaret Barton‐Burke:** Formal analysis (equal); investigation (equal); methodology (equal); resources (equal); writing – review and editing (equal). **Miranda Tan:** Conceptualization (lead); data curation (lead); methodology (lead); resources (lead); software (equal); supervision (lead); validation (lead); visualization (lead); writing – original draft (equal); writing – review and editing (lead).

## FUNDING INFORMATION

The authors' work in this study was supported in part by funding from the National Institutes of Health/National Cancer Institute (P30‐CA008748).

## CONFLICT OF INTEREST STATEMENT

S.V.C. has received travel reimbursement and an honorarium from Ipsen and is an advisor for Prostatype Genomics, unrelated to the present study.

## Data Availability

The data that support the findings of this study are available from the corresponding author upon reasonable request.
